# Maintenance of the Results of Stage II Lower Limb Lymphedema Treatment after Normalization of Leg Size

**DOI:** 10.1155/2017/8515767

**Published:** 2017-08-01

**Authors:** Jose Maria Pereira de Godoy, Henrique Jose Pereira de Godoy, Renata Lopes Pinto, Fernando Nestor Facio, Maria de Fatima Guerreiro Godoy

**Affiliations:** ^1^Cardiology and Cardiovascular Surgery Department, Faculty of Medicine of São José do Rio Preto (FAMERP), National Council for Research and Development (CNPq), São José do Rio Preto, SP, Brazil; ^2^Universidade Federal do Mato Grosso, Cuiabá, MT, Brazil; ^3^Research Group of Clínica Godoy, São José do Rio Preto, SP, Brazil; ^4^Faculty of Medicine of São José do Rio Preto (FAMERP) and Research Group of Clínica Godoy, São José do Rio Preto, SP, Brazil; ^5^Faculty of Medicine of São José do Rio Preto (FAMERP), São José do Rio Preto, SP, Brazil

## Abstract

**Objective:**

The aim of this study was to identify strategies to transfer responsibility of the maintenance of the results of lymphedema treatment to the patient.

**Methods:**

Maintenance of the reduction of edema was evaluated in a prospective clinical trial in patients with Stage II leg lymphedema. Twenty-one lymphedematous lower limbs were evaluated in Clínica Godoy in 2014 and 2016. The evaluation was done by volumetry at baseline and weekly thereafter for volume control. Patients wore Venosan® cotton 20/30 and 30/40 mmHg elastic compression stockings followed by a custom-made inelastic stocking made of grosgrain fabric. The Friedman test for multiple comparisons and Conover post hoc test were used for statistical analysis with an alpha error of 5%.

**Results:**

On comparing leg volume changes using the different types of stockings, the 20/30 mmHg elastic compression stockings failed in the first week to maintain the volume reductions but the 30/40 mmHg compression stockings did not allow significant increases in volume (*p* value > 0.05). During one week, the grosgrain stocking reduced leg volumes to baseline values (*p* value = 0.24).

**Conclusion:**

Higher compression of elastic stockings is better than lower compression but the inelastic grosgrain stocking is even better than both to maintain the results.

## 1. Introduction

Lymphedema is a chronic medical condition resulting from the accumulation of macromolecules in the interstitial space that leads to fluid retention. This condition may be due to congenital or acquired dysfunction of the lymphatic system [1].

Manual and Mechanical (RAGodoy®) Lymphatic Therapy cause the mobilization of macromolecules with reductions of the edema while compression mechanisms are key to maintaining the results [2]. The reduction of limb volume occurs due to loss of liquid by diuresis and redistribution of macromolecules and body fluid [3]. Thus, the association of drainage with restraint mechanisms, such as stockings or bandaging, can have a synergistic effect in reducing edema [4, 5].

Compression mechanisms can be elastic or inelastic; because of the differences in pressure at the skin-stocking interface, these characteristics affect treatment outcome [6]. Stockings have a great advantage because of their practicality; they can be donned and removed by the patient [5]. Bandages, on the other hand, require a specialized professional, as rarely patients are able to apply bandages by themselves [7–9].

Maintaining the results of lymphedema treatment is a challenge that requires continuous maintenance and frequent evaluations. Because of the chronicity of the disease, flexibility in the therapy and passing part of the responsibility of treatment to the patient are important to increase independence. The improvement in the quality of life of these patients including the physical, mental, and social domains is important and thus, stockings and constraint mechanisms can improve compliance and help maintain treatment results [10].

In recent years, Godoy & Godoy identified a type of textile named gorgurão in Brazil and grosgrain in English that satisfies the basic requisites for a compression garment that can be used to treat lymphedema. This fabric has different weights per square meter and thus adaptations need to be made depending on the quality of fabric used; however several studies show that it is suitable to treat lymphedema [11–13]. A soon-to-be-published study shows that it is even effective as monotherapy to reduce leg volume in patients with Stage II lymphedema.

The aim of this study was to identify strategies to maintain the results after total reduction of edema so that some of the responsibility of lymphedema treatment can be transferred to the patient.

## 2. Method

Twenty-one legs of 14 female and three male patients with lymphedema were evaluated in Clínica Godoy between 2013 and 2014. The mean age of the participants was 59.4 years.

Patients with Stage II lymphedema of the lower limbs, regardless of cause, were included if the size of the leg after treatment was within the normal range compared to the contralateral leg. Elderly patients were excluded, as were patients with chronic arterial insufficiency, morbid obesity, infections, joint immobility, or any other condition that would prevent the use of stockings. Patients were consecutively enrolled in order of arrival at the clinic.

The legs were evaluated by volumetry using the water displacement technique at baseline and weekly thereafter to control leg volume. Initially all patients wore Venosan 20/30 mmHg elastic compression stockings for four weeks. Subsequently, they used an inelastic custom-made stocking of grosgrain for one week. Finally, a Venosan 30/40 mmHg compression stocking was worn for four weeks.

## 3. Statistical Analysis

Descriptive statistics such as the mean, median, standard deviation, range, and upper and lower quartiles were used in the statistical analysis. The Friedman test for multiple comparisons and Conover post hoc test were used for statistical analysis with an alpha error of 5% being considered acceptable. This study was approved by the Research Ethics Committee of the Faculty of Medicine of São José do Rio Preto (FAMERP) number 052758/2014 (#CAAE: 32771414.8.40.54.15).

## 4. Results

Small and large variations in volume were detected during the study in part related to the type of compression stocking employed (Table 1).  Table 2 shows the descriptive statistics (mean and median, standard deviation, minimum and maximum variations, and upper and lower quartiles).  Table 3 shows the results of the Conover multiple comparisons test for volume changes using the Venosan 20/30 and 30/40 mmHg elastic stockings and the low-stretch grosgrain stocking.

**Figure 1:**
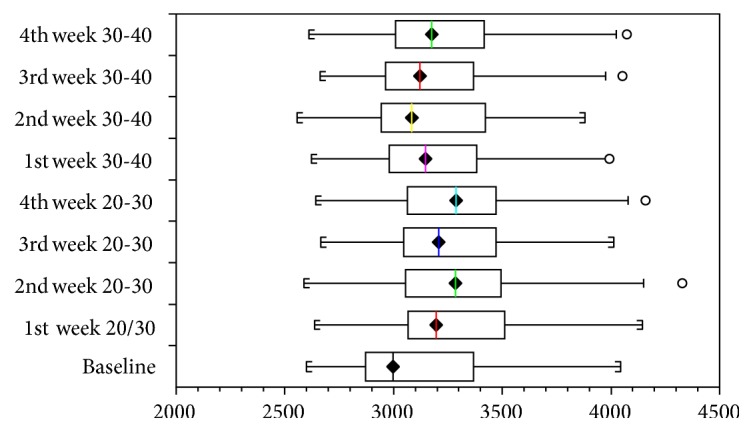
Volume changes in different weeks of treatment wearing 20/30 and 30/40 mmHg compression stockings.

The Friedman test identified significant differences in volumes (*p* value < 0.0001). When the final volumes related to the different types of stockings were compared using the Conover multiple comparisons test, the 20/30 mmHg compression stockings failed to maintain volume loss with a significant increase being seen within the first week (*p* value < 0.0001). However, on using 20/30 mmHg compression stockings, there were no significant volume differences comparing the first week with subsequent weeks (Table 3 and Figure 1).

**Figure 2:**
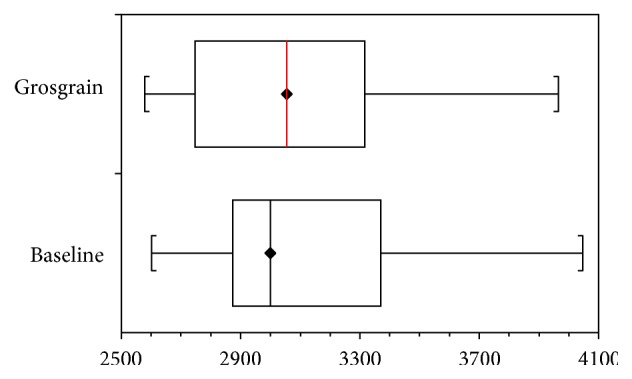
Interquartile range of volume changes between the baseline value and after one week of wearing grosgrain stockings.

When the respective weeks of 20/30 mmHg stockings are compared to 30/40 mmHg compression stockings, the latter was better to maintain treatment results (*p* values < 0.05). Use of the grosgrain stocking for one week reduced the sizes of the limbs to the baseline values (*p* value = 0.24; Figure 2).

## 5. Discussion

This study evaluated the strategy to maintain reductions in edema in patients submitted to treatment for lymphedema of the lower limbs using different elastic compression stockings and a low-stretch stocking made of grosgrain. Stockings with higher compression (30/40 mmHg) are superior to 20/30 mmHg compression stockings in the maintenance of treatment results. The 30/40 mmHg stocking preserved volume reductions over four weeks. However, the low-stretch stocking (grosgrain) is better than both elastic stockings in maintaining treatment results and even reducing the volume.

One of the challenges in the treatment of lymphedema is the maintenance of the results after total reduction of edema. It is known that lymphedema has no cure, so long-term maintenance therapy is critical throughout the lifetime of patients. The identification of alternative flexible approaches that improve patient compliance is important for therapeutic success. Recently, total reduction in the volume of edema ceased to be a major challenge in the treatment of these patients and consequently the maintenance of volume losses now requires more attention.

Elastic stockings, due to their convenience and availability, are a very good option to maintain volume reductions. However, they do not always preserve treatment results, perhaps due to a series of failures in the indication of an appropriate stocking.

Another aspect that draws attention in the clinical practice is about the use of stockings during the edema reduction phase. Manual and mechanical lymph drainage allow a rapid reduction of edema volume and the use of an elastic stocking has a synergistic effect in reducing edema during drainage. Therefore, the association of drainage with elastic stockings or bandages is essential at this stage. However, when reductions in limb size are more than 200 to 300 mL, the stockings fail to maintain further losses. In this phase, two stockings can be used, one on top of the other, or the stocking should be replaced.

Total reduction of edema is the goal of therapy; this should be achieved as soon as possible because it facilitates access of the patient to standard-sized stockings. Bandages can contribute to reductions in limb volume and are a further option [11].

The literature shows that stockings with high compression are the most suitable [10], but not all of the world's population has access to these stockings. Although this study shows that stockings with higher compression are better, lower compression can be used when there is no alternative.

Another important result of this study is related to grosgrain stockings, which currently are made by hand, but can be mass-produced. After total reduction of edema, patients themselves can make precise adjustments to the stocking as required. Grosgrain stockings can be used as monotherapy to normalize the size of the leg and maintain the results of lymphedema treatment. What limits their use is the time needed to don the stocking, which is about 10 minutes compared to 2 to 3 minutes for an elastic stocking. However, flexibility in using these stockings, according to need, will allow the patient to maintain normal or close to normal leg size.

In this study, the weekly volume variations were not large; the largest mean variation using elastic stockings was 158 mL with the 20/30 mmHg compression stockings compared to 63 mL with 30/40 mmHg compression. However, the use of a grosgrain stocking for one week reduced the leg volume by 159 mL on average, that is, below the initial volume, albeit not statistically significant.

Another key aspect is that the patient is able to control treatment, similar to other chronic diseases. It is essential for the professional to identify the time that each patient should return for assessments.

This study describes strategies to transfer part of the responsibility of lymphedema treatment to patients providing conditions for them to maintain the leg size within the normal range or close to it. Thus, the combination of grosgrain stockings with manual lymph drainage, when necessary, extends the therapeutic possibilities to control swelling. After complete reduction of the edema, maintenance therapy can be continued in accordance with the conditions and needs of each patient.

## 6. Conclusion

Elastic stockings with higher compression are superior to those of lower compression; however the inelastic grosgrain stocking is better than both, decreasing volume of lymphedema and maintaining the results. However, the practicality of elastic stockings makes them a good option; alternating elastic and low-stretch grosgrain stockings allows the patient to maintain volume reductions within the normal range or close to it.

## Figures and Tables

**Table 1:** Volumes of legs using 20/30 and 30/40 mmHg elastic and low-stretch compression stockings.

Baseline	20/30 mmHg compression stocking				Grosgrain	30/40 mmHg compression stocking			
	Week					Week			
	1st	2nd	3rd	4th		1st	2nd	3rd	4th
4046	4047	3957	3912	3949	3839	3879	3721	3890	3804
2601	2637	2590	2679	2643	2625	2671	2640	2670	2611
2993	3068	3041	3050	3043	2928	2989	2946	2932	2906
2775	2727	2775	2666	2676	2616	2622	2819	2729	2657
2998	3080	3132	3011	3087	3081	3107	2926	3103	2991
3434	3360	3468	3451	3453	3315	3399	3491	3370	3441
3152	3373	3351	3282	3289	3268	3186	3211	3277	3178
2971	3107	3363	3202	3369	2995	3098	2949	2975	3057
2996	3236	3254	3270	3263	2819	3112	3164	3106	3264
2811	3206	3348	3493	3489	2628	3149	3141	3368	3381
3672	3815	3895	3772	3944	3805	3787	3709	3892	3786
3748	3838	3782	3725	3685	3669	3692	3588	3588	3542
3103	3073	3015	3105	3122	3034	2865	3012	3045	3063
2640	2701	2730	2783	2755	2577	2669	2557	2662	2726
2935	3197	3279	3158	3238	3115	3270	2943	3222	3050
3305	3451	3286	3325	3374	3316	3367	3354	3204	3393
3261	3573	3519	3444	3380	3206	3322	3329	3312	3346
3114	3065	3318	3208	3292	3055	3055	3084	3123	3176
2999	3137	3069	3047	3117	2913	3158	3069	2952	3060
2793	2870	3086	3146	3018	2673	2975	3056	3122	3032
3915	4147	4328	4013	4159	3964	3994	3882	4054	4075

**Table 2:** Comparison between the initial volumes after treatment and volume variations over the weeks with patients using 20/30 and 30/40 mmHg elastic compression stockings and a grosgrain stocking.

Title	Mean	Median	Maximum	Minimum	Upper quartile	Lower quartile	Standard deviation
Baseline	3155.333	2999	4046	2601	3305	2935	403.4275
*20/30 mmHg elastic stocking*							
1st	3271.81	3197	4147	2637	3451	3068	419.9758
2nd	3313.619	3286	4328	2590	3468	3069	420.6742
3rd	3273.429	3208	4013	2666	3451	3050	368.7153
4th	3302.143	3289	4159	2643	3453	3087	398.5773

Grosgrain	3116.238	3055	3964	2577	3315	2819	420.7277
*30/40 mmHg elastic stocking*							
1st	3207.905	3149	3994	2622	3367	2989	382.0806
2nd	3171.00	3084	3882	2557	3354	2946	352.563
3rd	3218.857	3123	4054	2662	3368	2975	383.0062
4th	3216.143	3176	4075	2611	3393	3032	377.2265

**Table 3:** Multiple comparisons between weekly volume changes of the legs wearing different stockings.

Comparison	*p* value
Baseline versus 1st week 20/30 stocking	<0.0001
Baseline versus 2nd week 20/30 stocking	<0.0001
Baseline versus 3rd week 20/30 stocking	<0.0001
Baseline versus 4th week 20/30 stocking	<0.0001

Baseline versus 1st week 30/40 stocking	0.08
Baseline versus 2nd week 30/40 stocking	0.73
Baseline versus 3rd week 30/40 stocking	0.08
Baseline versus 4th week 30/40 stocking	0.15

1st week 20/30 versus 1st week 30/40 stocking	0.01
2nd week 20/30 versus 2nd week 30/40 stocking	0.001
3rd week 20/30 versus 3rd week 30/40 stocking	0.01
4th week 20/30 versus 4th week 30/40 stocking	0.0002

Baseline versus 1st week grosgrain stocking	0.24
